# Vaginal microbiota, genital inflammation, and neoplasia impact immune checkpoint protein profiles in the cervicovaginal microenvironment

**DOI:** 10.1038/s41698-020-0126-x

**Published:** 2020-08-03

**Authors:** Paweł Łaniewski, Haiyan Cui, Denise J. Roe, Dana M. Chase, Melissa M. Herbst-Kralovetz

**Affiliations:** 1 Department of Basic Medical Sciences, College of Medicine—Phoenix, University of Arizona, Phoenix, AZ USA; 2 UA Cancer Center, University of Arizona, Phoenix/Tucson, AZ USA; 3 Department of Obstetrics and Gynecology, College of Medicine–Phoenix, University of Arizona, Phoenix, AZ USA; 4 Valleywise Health Medical Center, Phoenix, AZ USA; 5 Dignity Health St. Joseph’s Hospital and Medical Center, Phoenix, AZ USA; 6 US Oncology, Phoenix, AZ USA

**Keywords:** Translational research, Next-generation sequencing, Cancer microenvironment, Cervical cancer, Immunotherapy

## Abstract

Emerging evidence suggests that the vaginal microbiota play a role in HPV persistence and cervical neoplasia development and progression. Here we examine a broad range of immune checkpoint proteins in the cervicovaginal microenvironment across cervical carcinogenesis and explore relationships among these key immunoregulatory proteins, the microbiota composition, and genital inflammation. First, we demonstrate that immune checkpoint molecules can be measured in cervicovaginal lavages. Secondly, we identify CD40, CD27, and TIM-3 to specifically discriminate cervical cancer from other groups and CD40, CD28, and TLR2 to positively correlate to genital inflammation. Finally, PD-L1 and LAG-3 levels negatively, whereas TLR2 positively correlate to health-associated *Lactobacillus* dominance. Overall, our study identifies immune checkpoint signatures associated with cervical neoplasm and illuminates the multifaceted microbiota-host immunity network in the local microenvironment. This study provides a foundation for future mechanistic studies and highlights the utility of cervicovaginal lavage profiling for predicting and monitoring response to cancer therapy.

## Introduction

Cervical cancer is the most common human papillomavirus (HPV)-related cancer and the fourth most common cancer in women worldwide with estimated 570,000 new cases and 311,000 deaths in 2018^1^. Over the last few decades, widespread use of screening methods, such as Papanicolaou test (cervical cytology) and HPV-based molecular tests, resulted in a dramatic decline in number of deaths related to cervical cancer in many high-income countries^2^. However, in lower income countries, the majority of women are diagnosed with advanced or metastatic carcinoma, which contributes to high mortality related to cervical cancer^1^. In addition, the death rates in the United States did not significantly change since 2007 (2.2 per 100,000 women) (https://www.cdc.gov/cancer/uscs/). The American Cancer Society estimates 13,170 new cases of cervical cancer cases and 4250 deaths in the United States in 2019^3^. The majority of these new cervical cancer cases occurred among women who had never or rarely been screened. It is also worth noting that 90% of cervical cancer cases could be also preventable through routine HPV vaccination for adolescent males and females^4^. Yet, the HPV vaccination coverage in the United States remains drastically lower (43% in 2017) compared with other recommended childhood vaccinations [e.g., 91% for measles, mumps, and rubella vaccine]^5^.

Standard treatments of cervical cancer include surgery, chemoradiation, or a combination of both, depending on the stage of cancer. Despite advances in screening and prevention, the 5-year overall survival for all stages of cervical cancer is 68%, whereas the 5-year overall survival for advanced cervical cancer is only 15%^6,7^. Introduction of an antiangiogenic agent (bevacizumab) to chemotherapy increased overall survival from 13 to 17 months for patients with persistent, recurrent, and metastatic cancer^8^. Yet, there is an urgent need to improve therapeutic outcomes, particularly for advanced or relapsed disease.

Recently, immune checkpoint inhibitors have emerged as a promising strategy for the treatment of advanced solid tumors, including cervical cancer^7^. Indeed, in 2018, the Food and Drug Administration approved pembrolizumab, a monoclonal antibody targeting the programmed cell death protein 1 (PD-1), for advanced cervical cancer with disease progression during or after chemotherapy^7^. Other immune checkpoint inhibitors currently under investigation for cervical cancer treatment include antibodies targeting programmed cell death ligand 1 (PD-L1) or cytotoxic T-lymphocyte antigen 4 (CTLA-4)^6,7^. However, the results from recent phase I/II clinical trials revealed that that overall response to anti-PD-1 or anti-PD-L1 therapies was low, ranging from 12.5 to 26%^9–11^. These clinical studies also demonstrated that the overall response rate was independent of PD-L1 expression, HPV status, or number of previous therapies^10,11^. These findings highlight that predictive biomarkers for patient responsiveness to immunotherapies are still urgently needed.

In recent years, the human gut microbiome has emerged as a potential modulator of responsiveness to immunotherapies. Indeed, clinical and animal studies demonstrated that specific enteric microorganisms mediate T-cell activation and increase T-cell priming and accumulation at the tumor site, which result in improved efficacy of immune checkpoint inhibitors^12–16^. These studies provide strong evidence of bidirectional relationships between the immune checkpoint proteins and the gut microbiome. Nevertheless, the possible relationships between immune checkpoint proteins in women with cervical neoplasm and the vaginal microbiome have not been investigated.

Vaginal microbiota in the majority of healthy premenopausal women is dominated by *Lactobacillus* species (*L. crispatus*, *L. gasseri*, *L. jensenii*, or *L. iners*), which protects the host against sexually transmitted infections, such as HPV^17^. To date, multiple epidemiological studies consistently demonstrated a decrease in *Lactobacillus* dominance and an increase in dysbiotic communities, characterized by overgrowth of diverse anaerobic microorganisms, in women with cervical dysplasia and cancer^18–21^. Two recent meta-analyses of available data strongly support a role of the vaginal microbiota in HPV persistence and cervical disease progression^22,23^. Our previous reports also illuminated the functional interplay between HPV, host defense mechanisms and the vaginal microbiota across cervical carcinogenesis^18,24,25^. Notably, we demonstrated that host factors in cervicovaginal lavages (CVL), including circulating cancer biomarkers, depend on genital inflammation and the vaginal microbiota composition^24^.

To better understand the biological mechanisms of cervical neoplastic disease, herein we investigated immune checkpoint protein profiles in CVL collected from women across cervical carcinogenesis in the context of the vaginal microbiota and genital inflammation. Our integrated approach uncovered the multifaceted interactions in the local microenvironment involving bacteria and mediators regulating host defense activation, which may be translated in future studies related to disease progression and/or efficacy of immunotherapies.

## Results

### Clinical and demographic information

In this cross-sectional study, we analyzed clinical samples, i.e., CVL and vaginal swabs, collected from 78 premenopausal, nonpregnant, Arizonan women with and without HPV infection and cervical neoplasia. Women were assigned to the following five groups: healthy HPV-negative controls (Ctrl HPV−; *n* = 18), HPV-positive controls (Ctrl HPV+; *n* = 11), women with low-grade intraepithelial lesions (LSIL; *n* = 12), high-grade intraepithelial lesions (HSIL; *n* = 27), and newly diagnosed invasive cervical carcinoma (ICC; *n* = 10). The classification to groups was based on the histology of biopsies, cytology (when histology was not available), and HPV genotyping as described previously^18^. Forty-seven percent of women were Hispanic and 53% women were non-Hispanic. Sixty-eight percent of women were overweight or obese [body mass index (BMI > 25) and the mean age across the groups was 38 ± 8 years. However, there were no significant differences among the groups with regard to Hispanic ethnicity (*P* = 0.15), BMI (*P* = 0.97), and age (*P* = 0.46).

### Local immune checkpoint proteins in cervicovaginal lavages

**Fig. 1:**
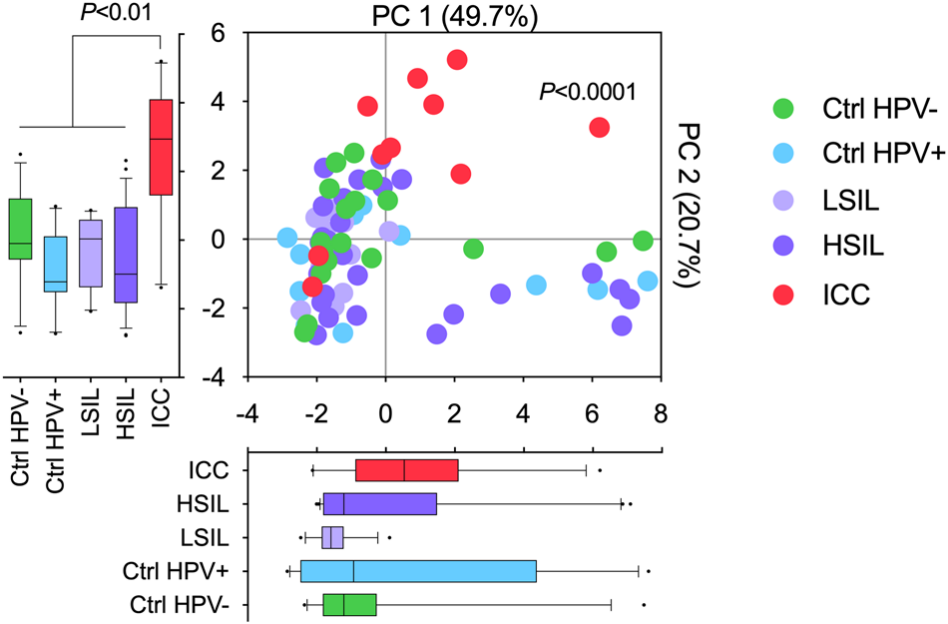
Cervical cancer patients exhibit distinct local immune checkpoint profiles. Immune checkpoint proteins are present in the local cervicovaginal microenvironment. Local protein profiles are distinct in cervical cancer patients compared with precancerous and control groups. Principal component analysis (PCA) of immune checkpoint protein profiles displayed along the first two principal components (PC), with each point representing a single sample colored according to disease group (*n* = 78). Box-and-whiskers plots shown along each PC axis represent the median and interquartile range with whiskers between 10th and 90th percentiles and indicate the distribution of samples along the given axis; dots indicate outliers. *P* value for the first two components was calculated using MANOVA, whereas *P* values for individual components were assessed using ANOVA.

To investigate immune checkpoint protein profiles in the local cervicovaginal microenvironment, we measured levels of 16 immune checkpoint proteins (see “Methods”) in CVL samples collected from women across cervical carcinogenesis. We were able to detect all protein targets. The principal component analysis (PCA), a data reduction method, was used to illustrate global immune checkpoint profiles among the groups (Fig. 1). We utilized the first two principal components (PC1 and PC2), which explained 70.4% of the variance in the data. Contributions of each immune checkpoint protein to PC1 and PC2 are shown in Supplementary Fig. S1. Multivariate analysis of variance (MANOVA) revealed significant differences among the groups (*P* < 0.0001). Subsequent pairwise comparisons showed that the ICC group is significantly different from all of the dysplasia (HSIL and LSIL) and control (Ctrl HPV− and Ctrl HPV+) groups (*P* ranging from 0.003 to 0.0001). Furthermore, when analyzing principal components separately, PC2, but not PC1, significantly varied between the ICC group when compared with all of the other groups (*P* ranging between 0.01 and <0.0001). This analysis demonstrated that immune checkpoint proteins can be detected in the local cervicovaginal microenvironment and ICC patients exhibit distinct immune checkpoint profiles compared with healthy HPV-negative women, HPV-positive women, and women with precancerous dysplasia.

### Immune checkpoint proteins and invasive cervical carcinoma

**Fig. 2:**
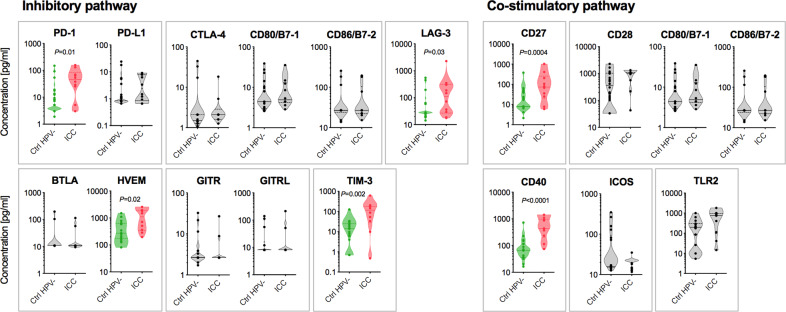
Local levels of immune checkpoint proteins are elevated in cervical cancer. Cervical cancer patients exhibit significantly elevated levels of immune checkpoint proteins from inhibitory and co-stimulatory pathways in cervicovaginal lavages compared with other groups. Violin plots show distribution of protein levels in ICC (*n* = 10) and Ctrl HPV− groups (*n* = 18). Dots indicate individual values for each sample and horizontal solid and dashed lines indicate median and first and third quartiles, respectively. Immune checkpoint proteins that form functional complexes were grouped together. *P* values were calculated using linear mixed effects models where group was the fixed effect and replicate was the random effect with Tukey adjustment. Immune checkpoint that reached significant differences (*P* < 0.05) between Ctrl HPV− and ICC are depicted in green and red, whereas immune checkpoint proteins that did not reached significant differences are depicted in gray. Levels of immune checkpoint proteins among all patient groups are included in Supplementary Fig. S2.

**Fig. 3:**
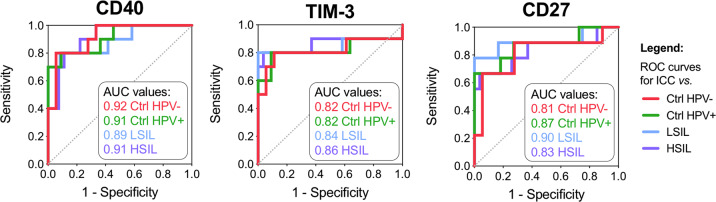
CD40, TIM-3, and CD27 discriminate cervical cancer from the other groups. CD40 is an excellent discriminator and TIM-3, CD27 are good discriminators for cervical cancer when compared with controls and precancerous dysplasia. The receiver operating characteristics (ROC) analysis comparing cervical cancer group (*n* = 10) to Ctrl HPV− (*n* = 18), Ctrl HPV+ (*n* = 11), LSIL (*n* = 12), or HSIL (*n* = 27) groups. ROC curves indicate specificity (*x* axis) and 1—sensitivity (*y* axis). Immune checkpoint proteins with the area under curve (AUC) greater than 0.7, 0.8, or 0.9 serve as fair, good or excellent discriminators, respectively. Only immune checkpoint proteins with AUC > 0.7 are depicted. ROC curves for all tested immune checkpoint proteins are included in Supplementary Fig. S3.

When we compared the levels of immune checkpoint proteins measured in CVL samples among the groups, we found that six out of sixteen targets were significantly elevated in women with ICC compared with the Ctrl HPV− group (*P* ranging from 0.03 to <0.0001) (Fig. 2) and other precancerous groups (Supplementary Fig. S2). Functionally, four immune checkpoint proteins, such as PD-1, lymphocyte activation gene 3 (LAG-3), herpesvirus entry mediator (HVEM) and T-cell immunoglobulin and mucin domain-containing 3 (TIM-3), are involved in the inhibitory pathways and two proteins, cluster of differentiation (CD) 27 and CD40, are involved in the co-stimulatory pathways. ICC patients also exhibited elevated levels of toll-like receptor 2 (TLR2) when compared with dysplasia and Ctrl HPV+ groups (*P* ranging from 0.03 to 0.003), but not when compared with Ctrl HPV− (Supplementary Fig. S2). None of the immune checkpoint proteins were significantly elevated in dysplasia groups when compared with controls (Supplementary Fig. S2). We also performed a receiver operating characteristic (ROC) curve analysis to evaluate the discrimination capacity of tested immune checkpoint proteins (Fig. 3 and Supplementary Fig. S3). Proteins with area under the curve (AUC), which plots the true positive rate (sensitivity) against the false positive rate (1—specificity), greater than 0.9 or 0.8 were considered as excellent or good discriminators, respectively. The analysis comparing ICC and Ctrl HPV− groups revealed three immune checkpoint proteins with excellent or good discriminatory properties, such as CD40 (AUC 0.92), TIM-3 (AUC 0.82), and CD27 (AUC 0.81). HVEM, PD-1, TLR2, and inducible T-cell co-stimulator (ICOS) exhibit only fair discrimination capabilities (AUC > 0.7), whereas other immune checkpoint proteins were poor discriminators (AUC < 0.7) (Supplementary Fig. S3). When comparing other groups, CD40, TIM-3, and CD27 also significantly discriminated ICC group from dysplasia and Ctrl HPV+ groups (AUC ranging from 0.82 to 0.91). These analyses demonstrated that specific immune checkpoint proteins are significantly and specifically elevated in women with ICC.

### Cervicovaginal immune checkpoint protein correlation network

**Fig. 4:**
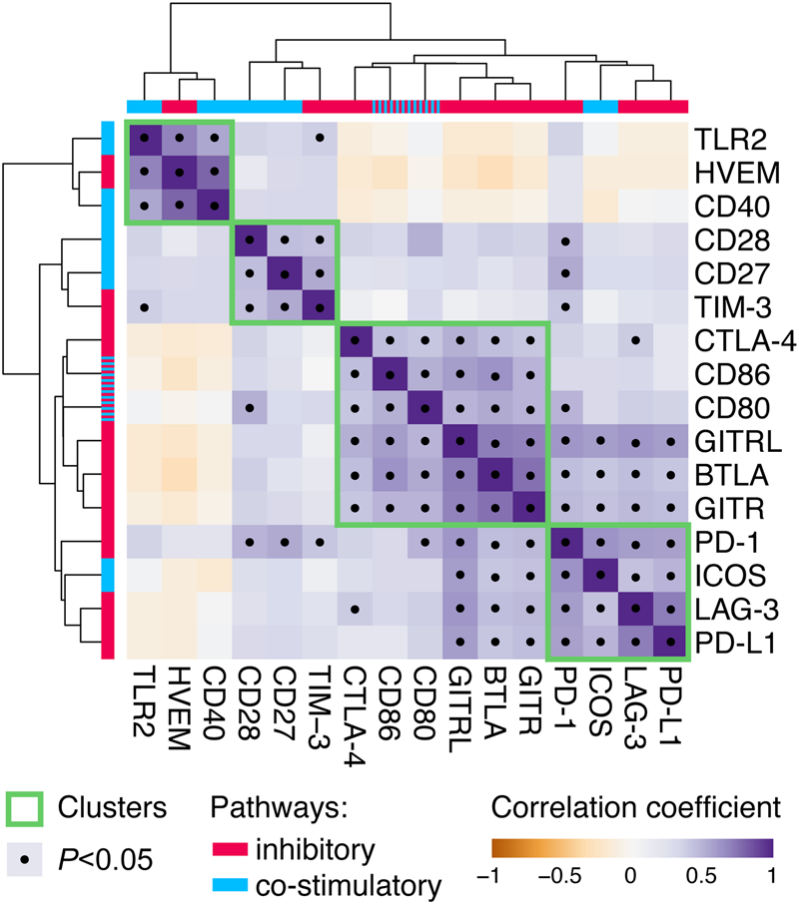
Correlation network of cervicovaginal immune checkpoint proteins. Levels of immune checkpoint proteins in the local microenvironment are strongly correlated to other immune checkpoint proteins. Correlation matrix of immune checkpoint proteins in the cervicovaginal lavages among all the patients (*n* = 78). Correlation coefficient (*ρ*) were calculated using Spearman’s rank correlation analysis. Hierarchical clustering of correlation coefficients was performed using ClustVis based on Euclidean distance and average linkage cluster algorithm. Purple- and yellow-shaded squares on the heatmap indicate positive and negative correlation, respectively. Clusters of proteins that strongly correlate to each other are depicted with green outlines. A bar below each dendrogram shows immune pathways utilized by immune checkpoint proteins. Significant correlations (*P* < 0.05) are indicated with black circles (•).

To identify potential biological networks among the immune checkpoint proteins, we computed a correlation matrix using the levels of immune checkpoints among all the patients regardless of the groups (Supplementary Fig. S4). Calculated Spearman’s coefficients (*ρ*) for each pair of immune checkpoint proteins were used in the hierarchical clustering analysis (HCA) and depicted as a heatmap (Fig. 4). The HCA revealed four major clusters of immune checkpoint proteins that significantly and positively correlated to each other: cluster 1 with CD40, HVEM, TLR2; cluster 2 with CD27, CD28, TIM-3; cluster 3 with B- and T-lymphocyte attenuator (BTLA), CD80, CD86, CTLA-4, glucocorticoid-induced tumor necrosis factor receptor-related protein (GITR), and GITR ligand (GITRL); and cluster 4 with ICOS, LAG-3, PD-1, and programmed cell death ligand 1 (PD-L1). All four clusters comprised of a mix of immune checkpoints belonging to both inhibitory and co-stimulatory pathways. Furthermore, the levels of immune checkpoint molecules in CVL samples positively and significantly correlated to the levels of their ligands, e.g., PD-1 levels correlated to PD-L1 levels (*ρ* = 0.523, *P* < 0.0001), CTLA-4 to CD80 (*ρ* = 0.402, *P* = 0.021) and CD86 (*ρ* = 0.456, *P* = 0.002), GITR to GITRL (*ρ* = 0.695, *P* < 0.0001). These data suggest that the production and/or secretion of immune checkpoint proteins is co-dependent and these molecules form a complex biological network involving both immune inhibitory and immune co-stimulatory pathways.

### Immune checkpoint proteins, inflammation, and microbiota

**Fig. 5:**
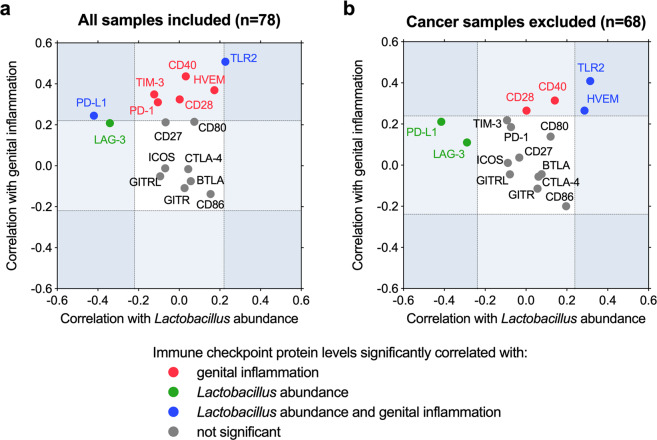
Key checkpoint protein correlate with *Lactobacillus* and genital inflammation. Scatterplots shows correlations of immune checkpoint proteins with *Lactobacillus* abundance (*x* axis) or genital inflammatory scores (*y* axis). Spearman’s correlation coefficients (*ρ*) were calculated using levels of immune checkpoint proteins for all samples (*n* = 78) (**a**) or without cancer samples (*n* = 68) (**b**). *Lactobacillus* abundance was determined by 16S rRNA gene sequencing. Levels of seven cytokines (IL-1α, IL-1β, IL-8, MIP-1β, MIP-3α, RANTES, and TNFα) were evaluated in CVLs and the patients were assigned a genital inflammatory score (0–7) based on whether the level of each cytokine was in the upper quartile. Significant correlations (*P* < 0.05) between immune checkpoint proteins and genital inflammatory scores, *Lactobacillus* abundance or both are indicated with red, green, and blue circles, respectively.

Previously we demonstrated that the severity of cervical neoplasia is linked to genital inflammation and vaginal microbiota composition^18^, herein we extend these findings and investigated relationships between the levels of immune checkpoint proteins, genital inflammatory scores, and *Lactobacillus* abundance. The genital inflammation scoring system was described previously^18^. Briefly, levels of seven cytokines, including interleukin (IL)-1α, IL-1β, IL-8, macrophage inflammatory protein (MIP)-1β, MIP-3α, regulated on activation, normal T cell expressed and secreted (RANTES), and tumor necrosis factor (TNFα), were evaluated in CVL samples and women were assigned a genital inflammatory score (0–7) based on whether the level of each cytokine was in the upper quartile. These cumulative inflammatory scores also reflected elevated levels of other pro-inflammatory immune mediators tested, but not included in the score, therefore this scoring system can accurately reflect genital inflammation^18^. Relative abundance of *Lactobacillus* and other vaginal genera was determined previously using 16S rRNA gene sequencing and DNA extracted from vaginal swabs^18^. The Spearman’s correlation coefficients (*ρ*) were calculated for each immune checkpoint protein (in all patients regardless of disease group) and genital inflammatory scores, as well as, for each immune checkpoint protein and *Lactobacillus* abundance (Supplementary Figs. S5–8). The computed correlation coefficients were depicted as a scatterplot showing the correlation with inflammatory scores on *y* axis and correlation with *Lactobacillus* abundance on *x* axis (Fig. 5a). The analysis demonstrated that several immune checkpoint proteins (CD28, CD40, HVEM, PD-1, PD-L1, TIM-3, and TLR2) positively (*ρ* ranging from 0.245 to 0.508) and significantly (*P* ranging from 0.03 to <0.001) correlated to genital inflammation. Two of these immune checkpoint proteins also correlated to *Lactobacillus* abundance: PD-L1 in a negative relationship (*ρ* = −0.420; *P* < 0.001) and TLR2 in a positive relationship (*ρ* = 0.227; *P* = 0.046). However, LAG-3 negatively correlated to *Lactobacillus* abundance (*ρ* = −0.341; *P* = 0.002), but did not significantly correlate to genital inflammation.

To better understand the relationships independent of cancer, we also performed the correlation analysis by excluding ICC samples (Fig. 5b). In this analysis, CD28 (*ρ* = 0.265; *P* = 0.029) and CD40 (*ρ* = 0.314; *P* = 0.009) still positively correlated to genital inflammation, whereas HVEM and TLR2 positively correlated to both genital inflammation and *Lactobacillus* abundance (*ρ* ranging from 0.265 to 0.409; *P* ranging from 0.029 to 0.001). PD-L1 did not correlate to genital inflammation; however, it still exhibited a negative correlation to *Lactobacillus* abundance (*ρ* = −0.415; *P* = 0.001), similarly to LAG-3 (*ρ* = −0.289; *P* = 0.018). These analyses revealed associations between the local levels of several immune checkpoint proteins with the vaginal microbiota composition and genital inflammation, which was independent of cancer.

**Fig. 6:**
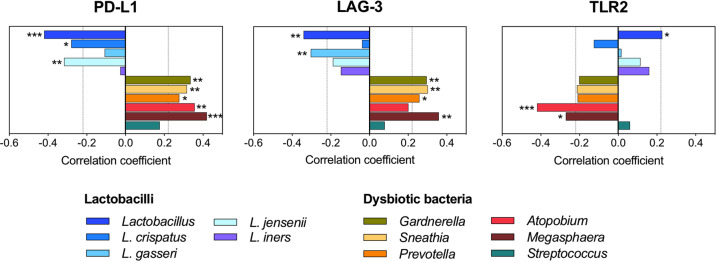
PD-L1, LAG-3, and TLR2 correlate to most abundant vaginal bacterial species. PD-L1 and LAG-3 negatively correlated to *Lactobacillus* species and positively correlated to dysbiotic bacteria, whereas TLR2 negatively correlated to dysbiotic bacteria and positively correlated to lactobacilli. Spearman correlation coefficients (*ρ*) were calculated using levels of immune checkpoint proteins for all samples (*n* = 78) and relative abundances/levels of vaginal bacterial taxa. Relative abundances of most prevalent vaginal genera (i.e., *Lactobacillus*, *Gardnerella*, *Sneathia*, *Prevotella*, *Atopobium*, *Megasphaera*, and *Streptococcus*) were determined by 16S rRNA gene sequencing. Relative levels of vaginal *Lactobacillus* species (*L. crispatus*, *L. gasseri*, *L. jensenii,* and *L. iners*) were determined by quantitative real-time PCR assays. *P* values are indicated with asterisks (***P < 0.001; **P < 0.01; *P < 0.05).

To further explore the relationship between PD-L1, LAG-3 and TLR2 and vaginal microbiota composition, we performed additional correlation analyses using the relative abundance of the most prevalent vaginal bacterial taxa detected in our samples, including four predominant vaginal *Lactobacillus* species (*L. crispatus*, *L. gasseri*, *L. jensenii*, and *L. iners*), as well as, bacteria associated with vaginal dysbiosis (*Gardnerella*, *Sneathia*, *Prevotella*, *Atopobium*, and *Megasphaera*) and vaginal pathobionts (*Streptococcus*) (Fig. 6). The analysis revealed significant correlations between the PD-L1, LAG-3, TLR2 levels, and abundance/levels of multiple vaginal bacterial taxa. The directionality of these correlations varied for health-associated *Lactobacillus* species versus bacteria associated with vaginal dysbiosis for each of the immune checkpoint protein. For instance, PD-L1 negatively correlated to *L. crispatus* (*ρ* = −0.280; *P* = 0.014) and *L. jensenii* (*ρ* = −0.317; *P* = 0.005) and positively correlated with dysbiosis-associated bacteria *Gardnerella*, *Sneathia*, *Prevotella*, *Atopobium*, and *Megasphaera* (*ρ* ranging from 0.278 to 0.420; *P* ranging from 0.015 to 0.001), whereas LAG-3 negatively correlated to *L. gasseri* (*ρ* = −0.304; *P* = 0.007) and positively correlated to *Gardnerella*, *Sneathia*, *Prevotella*, and *Megasphaera* (*ρ* ranging from 0.259 to 0.359; *P* ranging from 0.023 to 0.001). On the other hand, TLR2 positively correlated to *Lactobacillus* (*ρ* = 0.227; *P* = 0.046), and negatively correlated to *Atopobium* (*ρ* = −0.420; *P* < 0.001) and *Megasphaera* (*ρ* = −0.271; *P* = 0.017). However, with TLR2 we did not observe any significant correlations to specific vaginal *Lactobacillus* species. We did not observe any significant correlations of PD-L1, LAG-3, or TLR2 to *Streptococcus* (vaginal pathobiont) or *L. iners* (intermediate *Lactobacillus* species associated with the transition to vaginal dysbiosis). This analysis further confirmed the observed strong associations (Fig. 5) between the local levels of immune checkpoint proteins and the vaginal microbiota composition.

## Discussion

The immune system plays a vital role in the control of HPV infection, but the virus can evade immune surveillance^26^. Thus, the interplay between HPV-infected host cells and the local immune microenvironment determines the course of neoplastic disease in cervical carcinogenesis. The human microbiome can modulate immune responses within this environment, including critical components of antitumor immunity, and thereby contributes to the therapeutic activity of immune checkpoint inhibitors^15,16,27,28^. However, the crosstalk between the local vaginal microbiome and immune microenvironment during cervical carcinogenesis is still not well understood. In this study, we examined a broad range of immune checkpoint proteins in the local cervicovaginal microenvironment in women with and without cervical neoplasm and explored relationships among these key immunoregulatory proteins, the vaginal microbiota composition and genital inflammation.

Herein, we have demonstrated that immune checkpoint proteins can be measured in CVL, which indicate the presence of soluble forms of these proteins in the cervicovaginal microenvironment. The soluble forms of numerous immune checkpoint proteins have been reported and shown to be diffused into the serum^29,30^. Notably, these secreted forms may function distinctly compared with membrane-bound forms^31^. However, the role of these soluble immune checkpoint proteins in cervical carcinogenesis have not been comprehensively examined. To date, expression of immune checkpoint proteins in cervical cancer patients have been mostly evaluated in tissues samples using immunohistochemical staining. Compared with biopsy, the collection of CVL is a minimally invasive, low-cost and relatively easy procedure to perform. In our study, we identified four immune inhibitory checkpoint molecules (PD-1, HVEM, LAG-3, and TIM-3) and two immune co-stimulatory checkpoint molecules (CD27 and CD40) to be significantly elevated in CVL collected from patients with cervical cancer compared with healthy women or women with precancerous dysplasia. Previous reports also demonstrated that CD40, PD-1, and TIM-3 are overexpressed in cervical carcinoma tissues compared with normal cervix^32–34^, which is consistent with our CVL findings. On the other hand, we did not observe elevated levels of PD-L1, which has been previously shown to be overexpressed in cervical cancer tissue^33^. Future studies are needed to comprehensively evaluate whether levels of immune checkpoint proteins in tissue and CVL samples correlate to each other; nonetheless, proteins detected in CVL have a great potential to be exploited for monitoring responses to therapies, as well as used as predictive or prognostic biomarkers in women with cervical cancer and other gynecological conditions.

Biomarker discovery analysis revealed that three immune checkpoint molecules, specifically CD40, CD27, and TIM-3, exhibit excellent or good discriminatory properties for cervical carcinoma compared to healthy controls and dysplasia. High specificity and sensitivity of secreted CD40, CD27, and TIM-3 for cervical cancer make these proteins potential therapeutic targets for immunotherapy with radiation. Intriguingly, early phase clinical trials attempted to employ monoclonal antibodies targeting CD40, CD27, or TIM-3 for treatment of advanced solid tumors (including HPV-related head and neck cancer), mostly in combination with PD-1/PD-L1 inhibitors^35–38^. The rationale for combination therapies is to couple therapeutics with different mechanisms of action in order to attain additive or synergistic effect on antitumor response^39^. For example, PD-1/PD-L1 or TIM-3 blockade reverse T-cell exhaustion, whereas therapies with CD40 and CD27 agonists lead to activation of dendritic cells and subsequent T-cell priming^39^. Combination therapies with monoclonal antibodies targeting CD40, CD27, or TIM-3 might be a potential approach to achieve better therapeutic outcomes in advanced and recurrent cervical cancer. Furthermore, immune checkpoint proteins detected in CVL samples, for example TIM-3, might be putatively used as biomarkers for poor prognosis and/or response to therapies for cervical cancer. A previous study showed that cervical cancer patients with high TIM-3 expression in cancerous tissues had a significantly higher metastatic potential, advanced cancer grades, and shorter survival than those with low TIM-3 expression^34^. TIM-3 has been also associated with advanced disease, recurrence, and decreased survival in other solid tumors, including kidney^40^, liver^41^, gastric^42^, and colorectal cancers^43^. The prognostic value of CVL levels of TIM-3 and other immune checkpoint molecules in patients with cervical cancer warrants further investigation.

In recent years, the human microbiome has emerged to be related, not only to development and progression of many malignancies^44^, but also to the effectiveness of immunotherapies^45^. In addition, multiple reports strongly support the role of vaginal microbiota in cervical carcinogenesis through complex interactions with HPV and the host immunity^18–25,46^. Previously, we and others demonstrated that the severity of cervical neoplasm is linked to changes in the vaginal microbiota composition, specifically depletion of health-associated *Lactobacillus* spp. and overgrowth of anaerobic bacteria associated with dysbiosis, including *Gardnerella vaginalis*, *Prevotella* spp., *Atopobium vaginae* and *Sneathia* spp., *Megasphaera* spp., and others^18,19,21^. Previously, we also revealed that changes in the vaginal microbiota composition across cervical carcinogenesis are associated with evidence of genital inflammation, altered proliferation, and apoptosis, angiogenesis, hormonal imbalance, and metabolic dysregulation^24,25^, all hallmarks of cancer. We hypothesize that these features of local microenvironment might suppress antitumor immunity and favor disease progression. In the presented study we extended these findings and illuminated that secreted immune checkpoint molecules, which are critical immunoregulatory proteins involved in cancer immune evasion, also relate to level genital inflammation and the vaginal microbiota composition.

We observed the complex network among immune checkpoint molecules, as many of them correlate to other immune checkpoint proteins. This can reflect previous observations that these proteins are frequently co-expressed on multiple cell types, including T cells, antigen-presenting cell, and/or malignant cells^47^. We also observed that several immune checkpoint proteins positively correlate to genital inflammation, which was highly elevated in cancer patients compared with other groups^18^. To attenuate the effect of cancer on these relationships, we excluded cancer samples from our subsequent analysis. We still observed significant correlation of two immune co-stimulatory molecules, CD40 and CD28, with the level of genital inflammation across patients with HPV infection, cervical dysplasia, and healthy controls. CD40/CD40 ligand (CD40L) pathway is known to play a critical role in host immunity against intracellular bacterial and viral pathogens by promoting Th-1-dominant responses^48^. Intriguingly, HPV infection has been shown to dysregulate CD40/CD40L pathway in skin epithelial cells^49^. The role of CD28 in controlling HPV infection is not well understood. However, a report on chlamydial infection in mouse model revealed that co-stimulation via CD40, but not CD28, was required for the development of protective immunity; however, CD28 co-stimulation contributed to inflammatory pathologies in the murine reproductive tract^50^. The function of immune co-stimulatory molecules in HPV-mediated immune evasion should be comprehensively investigated in future studies.

In the presented study, we also uncover relationships between specific immune checkpoint molecules and the vaginal microbiota composition. We identified two proteins, namely PD-L1 and LAG-3, to be associated with dysbiotic *Lactobacillus*-depleted microbiota. Previously, the evidence of interplay between microbiota and immune checkpoints have been demonstrated in the gut. Specific intestinal microorganisms, including *Bifidobacterium* spp., have been associated with favorable antitumor immune responses to immunotherapies targeting PD-1/PD-L1 axis in both preclinical animal models and patients with cancer^15,16,27,28^. Interestingly, PD-1 has been shown to impact IgA production and consequently the microbiota composition in the gut. Specifically, depletion in beneficial *Bifidobacterium* spp. and increase in abundance of dysbiotic *Enterobacteriaceae*, have been observed in PD-1-deficient mice^51^. The effect of PD-L1 or LAG-3 on microbial communities in the gut or other mucosal sites has not been thoroughly investigated. However, a recent mouse study highlighted that the lung microbiota induces transient expression of PD-L1 and regulatory T cells in the lungs, which promotes tolerance to allergens in neonates^52^. Another study using antibiotic-treated mice, demonstrated that the gut microbiota promotes intraepithelial lymphocytes which plays a critical role in suppressing autoimmunity in the central nervous system via a LAG-3-dependent mechanism^53^.

In the presented study, we also identified TLR2 to be strongly associated with *Lactobacillus* dominance, but also with elevated genital inflammation. Toll-like receptors are critical components of the innate immune system in the female reproductive tract^54^. TLR2 recognizes bacterial lipoproteins and viral proteins; thus, plays a key role in defense against both bacterial and viral infections^55^. A previous prospective clinical study revealed that high expression levels of nucleic acid-sensing TLRs (TLR3 and TLR7), but not TLR2, were significant predictors of HPV clearance^56^. However, another study revealed that women with regression of precancerous cervical dysplasia exhibited higher levels of TLR2 and TLR7 in endocervical cells compared with women with disease persistence or progression^57^, suggesting an important role of TLR2 in cervical carcinogenesis. Intriguingly, some commensal microorganisms have been shown to utilize the TLR2 signaling pathway to modulate inflammation^58^, as well as, carcinogenesis in the gut^59^. Specifically, *B. fragilis* inhibited tumor development in a mouse model of colitis-associated colorectal cancer^59^. This protective function relied on production of polysaccharide A and was dependent on TLR2 signaling^59^. Similarly, there is evidence that vaginal *Lactobacillus* species might also utilize TLR2 signaling to modulate immunity in the cervicovaginal microenvironment. An in vitro study demonstrated that *L. crispatus* can facilitate differentiation of monocytic precursor cells into dendritic-like cells through activation of TLR2/6 by peptidoglycans, a bacterial cell component^60^. Thus, the presence of *L. crispatus* in the local microenvironment might directly contribute to antiviral and antitumor immunity. Our findings provide a basis for further investigations of a role of vaginal microbiota in modulating antitumor host responses through TLR signaling.

**Fig. 7:**
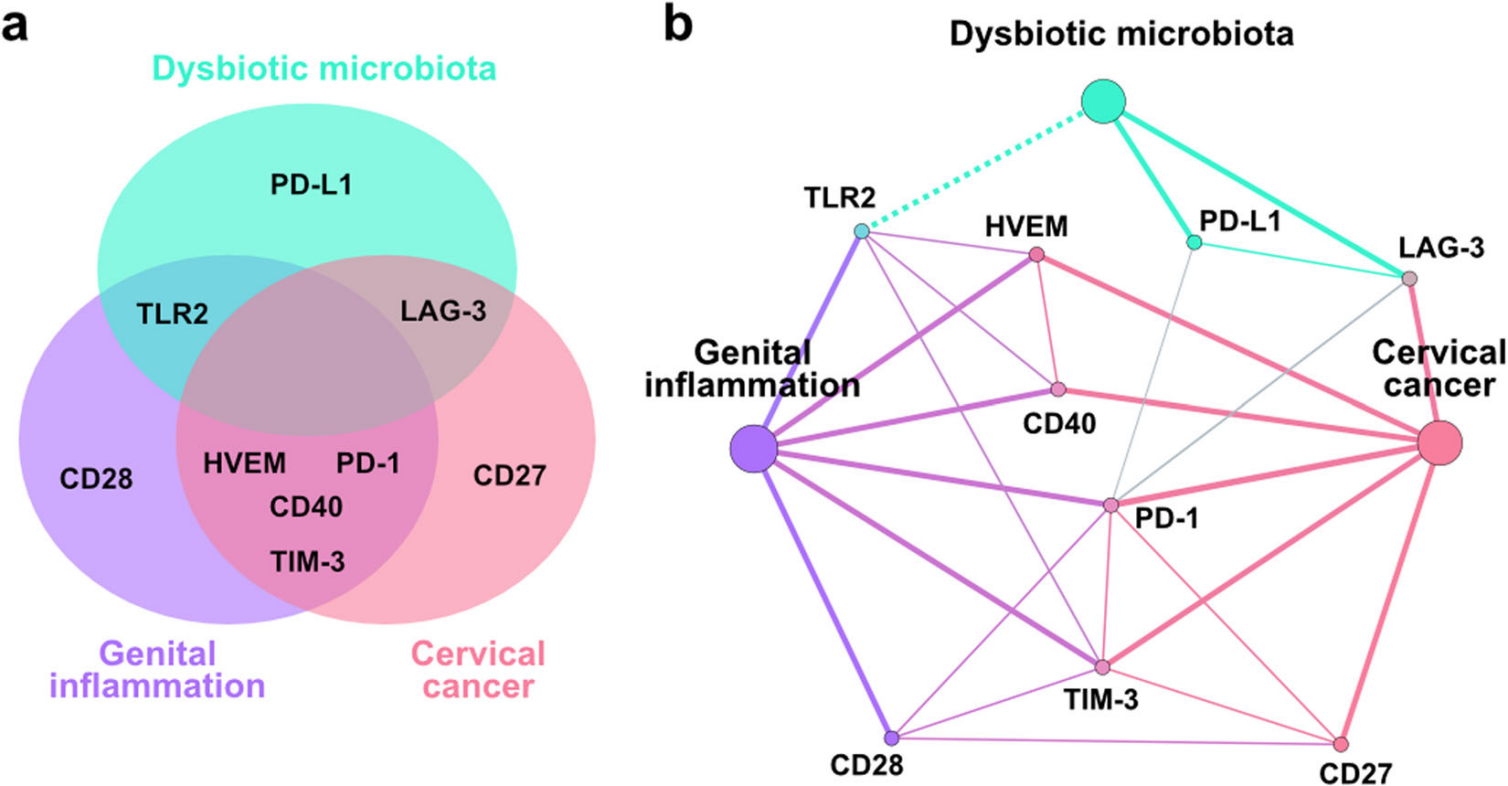
A complex host-microbe network in the cervicovaginal microenvironment. Venn (**a**) and network (**b**) diagrams summarize the results of this study and depict immune checkpoint proteins significantly elevated in patients with invasive cervical carcinoma when compared with HPV-negative controls (indicated in pink); immune checkpoint proteins significantly correlated to genital inflammatory scores (indicated in purple); and immune checkpoint proteins significantly correlated to vaginal microbiota structure (indicated in green). The network diagram also shows correlations of immune checkpoint proteins to other immune checkpoint proteins. Solid and dotted line indicate positive or negative relationships, respectively.

In summary, we identified immune checkpoint signatures associated with cervical carcinogenesis and illuminated the multifaceted microbiota-host immunity network in the local microenvironment (Fig. 7). Elevated levels of CD40, HVEM, PD-1, and TIM-3 connected cervical carcinoma to genital inflammation, whereas LAG-3 connected carcinoma to dysbiotic microbiota and TLR2 bridged genital inflammation and *Lactobacillus* dominance. None of the immune checkpoint proteins tested related to all features of cancer, inflammation, and microbiota; however, multiple immune checkpoint proteins correlated to each other, relating all features together, which highlights the complex interactions between host, HPV, and microbiota during cervical carcinogenesis.

Limitations of our study include a relatively small sample size and a cross-sectional study design, which allows one to demonstrate associations, but not causation. Due to the given sample size, we were not able to adjust the data for all potential confounders. We also acknowledge that the correlation values, indicate moderate associations between variables. However, we used rigorous inclusion and exclusion criteria, which were described in detail previously^18^. Future studies with a larger sample size and longitudinal study designs are needed to extend our findings. Mechanistic studies utilizing in vitro and in vivo models are also required to illuminate biological mechanisms of the complex interplay between host immunity, the vaginal microbiota, and cancer therapies.

In conclusion, we showed that immune checkpoint molecules can be detected in the cervicovaginal microenvironment in women across cervical carcinogenesis and, notably, that the levels of these molecules depend on genital inflammation and the vaginal microbiota composition. In the future, these or other protein targets, measured in CVL, might be utilized as prognostic or predictive biomarkers for cervical cancer and other gynecologic conditions. These relationships in the cervicovaginal microenvironment, similarly to those observed in the gut, may impact responsiveness to immunotherapy and/or immune-related toxicities, particularly in patients with gynecologic cancer, and should be explored in future clinical studies.

## Methods

### Study population and sample collection

Seventy-eight premenopausal, nonpregnant women were recruited at three clinical sites in Phoenix, AZ: St. Joseph’s Hospital and Medical Center, University of Arizona Cancer Center and Valleywise Health Medical Center. All participants provided informed written consent and all research and related activities involving human subjects were approved by the Institutional Review Boards at each participating site. The participants were grouped as follows: Ctrl HPV− (*n* = 18), Ctrl HPV+ (*n* = 11), LSIL (*n* = 12), HSIL (*n* = 27), and ICC (*n* = 10). Only patients with newly diagnosed ICC were included in the study. Classification of patients into the five groups was based on histology of biopsy samples or cytology (if histology was not available). Detailed exclusion criteria were described previously^18^. CVL and vaginal swabs were collected by a physician and processed as described previously for microbiome and immunoproteome analyses^18^. Briefly, CVLs were collected using 10 ml of sterile 0.9% saline, immediately placed on ice and frozen at −80 °C for further analysis. DNA was extracted from vaginal swabs using the PowerSoil DNA Isolation Kit (MOBIO Laboratories, Carlsbad, CA). 16S rRNA of V4 region was performed by the Second Genome (San Francisco, CA) using extracted DNA and V4-specific primers. Relative levels of *Lactobacillus* species were determined by quantitative real-rime PCR using species-specific and pan-bacterial Taqman® probes. HPV status was determined using DNA from collected vaginal swabs and the Linear Array HPV Genotyping Test (Roche, Indianapolis, IN) to classify patients into Ctrl HPV− and Ctrl HPV+ groups^18^. Demographic data were collected from surveys and/or medical records. Statistical differences in the demographic variables between patient groups were tested using an analysis of variance (ANOVA) for continuous variables and Fisher’s exact test for categorical variables. All statistical analyses were performed using SAS 9.4 (SAS Institute, Cary, NC), unless otherwise stated.

### Measurement of local immune checkpoint proteins

Levels of sixteen immune checkpoint proteins (BTLA, CD27, CD28, CD40, CD80/B7-1, CD86/B7-2, CTLA-4, GITR, GITRL, HVEM, ICOS, LAG-3, PD-1, PD-L1, TIM-3, and TLR2) were determined in CVL samples using the MILLIPLEX MAP® Human Immuno-Oncology Checkpoint Protein Magnetic Bead Panel (Millipore, Billerica, MA) in accordance with the manufacturer’s protocol. Data were collected with a Bio-Plex® 200 instrument and analyzed using Manager 5.0 software (Bio-Rad, Hercules, CA). A four-parameter logistic regression curve fit was used to determine the concentration. All samples were assayed in duplicate. The concentration values below the detection limit were substituted with 0.5 of the minimum detectable concentration provided in manufacturer’s instructions. The natural logarithm (ln) transformation was applied to normalize the data.

### Principal component analysis

The PCA was performed to reduce the observed variables into a smaller number of principal components (artificial variables) that will account for most of the variance in the observed variables. For the first two components, the difference among groups was assessed using the MANOVA model. If the overall difference was significant (*P* < 0.05), pairwise comparisons with Tukey adjustment were performed. The statistical differences for individual components were assessed using ANOVA.

### Linear mixed effects model

The statistical differences in the concentration among the patient groups were tested using a linear mixed effects model where the group was a fixed effect and the replicate was the random effect. If the overall difference was significant (*P* < 0.05), paired tests were performed with Tukey adjustment.

### Receiver operating characteristics analysis

The ROC analysis was performed to identify immune checkpoint proteins that discriminate specific patient groups. The mean levels of immune checkpoint proteins for each patient were used in the analyses. The strength of the discriminators was measured with AUC values. Proteins with AUC greater than 0.7, 0.8, or 0.9 were considered as fair, good, or excellent discriminators, respectively. The analysis was performed using Prism 5.0 software (GraphPad, San Diego, CA).

### Correlation analyses

The Spearman’s rank correlation analyses were performed to investigate association of immune checkpoint proteins to other immune checkpoint proteins, vaginal microbiota, and genital inflammation. Spearman’s rank correlation coefficients (*ρ*) were computed using log-transformed levels of immune checkpoint proteins among all patients (*n* = 78), relative abundance of vaginal bacterial genera (i.e., *Lactobacillus*, *Gardnerella*, *Prevotella*, *Sneathia*, *Atopobium*, *Megasphaera*, and *Streptococcus*), relative levels of vaginal *Lactobacillus* species (*L. crispatus*, *L. gasseri*, *L. jensenii*, and *L. iners*), and genital inflammatory scores. Relative abundances of vaginal taxa were determined by 16S rRNA gene sequencing and relative levels of *Lactobacillus* species were determined by quantitative real-rime PCR using species-specific and pan-bacterial Taqman® probes and described previously^18^. Genital inflammatory scoring was also described previously^18^. Briefly, levels of seven cytokines (IL-1α, IL-1β, IL-8, MIP-1β, MIP-3α/CCL20, RANTES, and TNFα) were evaluated in CVLs and the patients were assigned a genital inflammatory score (0–7) based on whether the level of each cytokine was in the upper quartile. *P* values < 0.05 were considered significant.

### Hierarchical clustering analysis

The HCA was performed to show relationships of immune checkpoint proteins to other immune checkpoint proteins. Clustering of computed correlation coefficients (*ρ*) was performed using ClustVis and based on Euclidean distance between rows and columns and average linkage cluster algorithm^61^.

### Reporting summary

Further information on research design is available in the Nature Research Reporting Summary linked to this article.

## Supplementary information

Supplementary figures S1-S8

Reporting summary

## Data Availability

Sequence data that supports the findings of this study have been deposited in SRA (PRJNA518153).
