# Supplementary material for: Vaginal microbiota, genital inflammation, and neoplasia impact immune checkpoint protein profiles in the cervicovaginal microenvironment
Source: NPJ Precis Oncol. 2020 Aug 3;4:22. doi: 10.1038/s41698-020-0126-x (PMC7398915; doi:10.1038/s41698-020-0126-x)
Supplement: Supplementary file 1 — Supplementary figures S1-S8 [file 41698_2020_126_MOESM1_ESM.pdf]

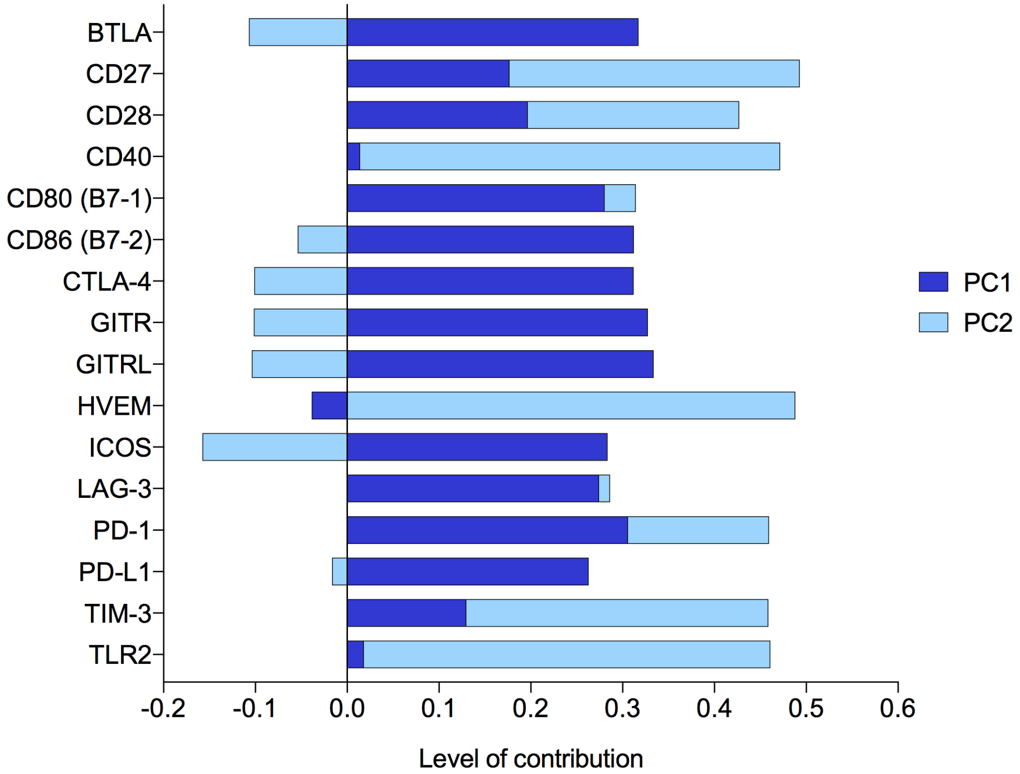

Supplementary Figure 1. Contribution of immune checkpoint proteins in the principal component analysis (PCA). Stacked bar plots show contribution of each immune checkpoint protein to principal 1q principal component (PC2), which explain 49.7% and 20.7% of the variance in the data, respectively. CD80, LAG-3 and PD-L1 levels contribute mostly to PC1 and CD40, HVEM and TLR2 levels contribute mostly to PC2, whereas the other immune checkpoint proteins contribute to both PC1 and PC2.

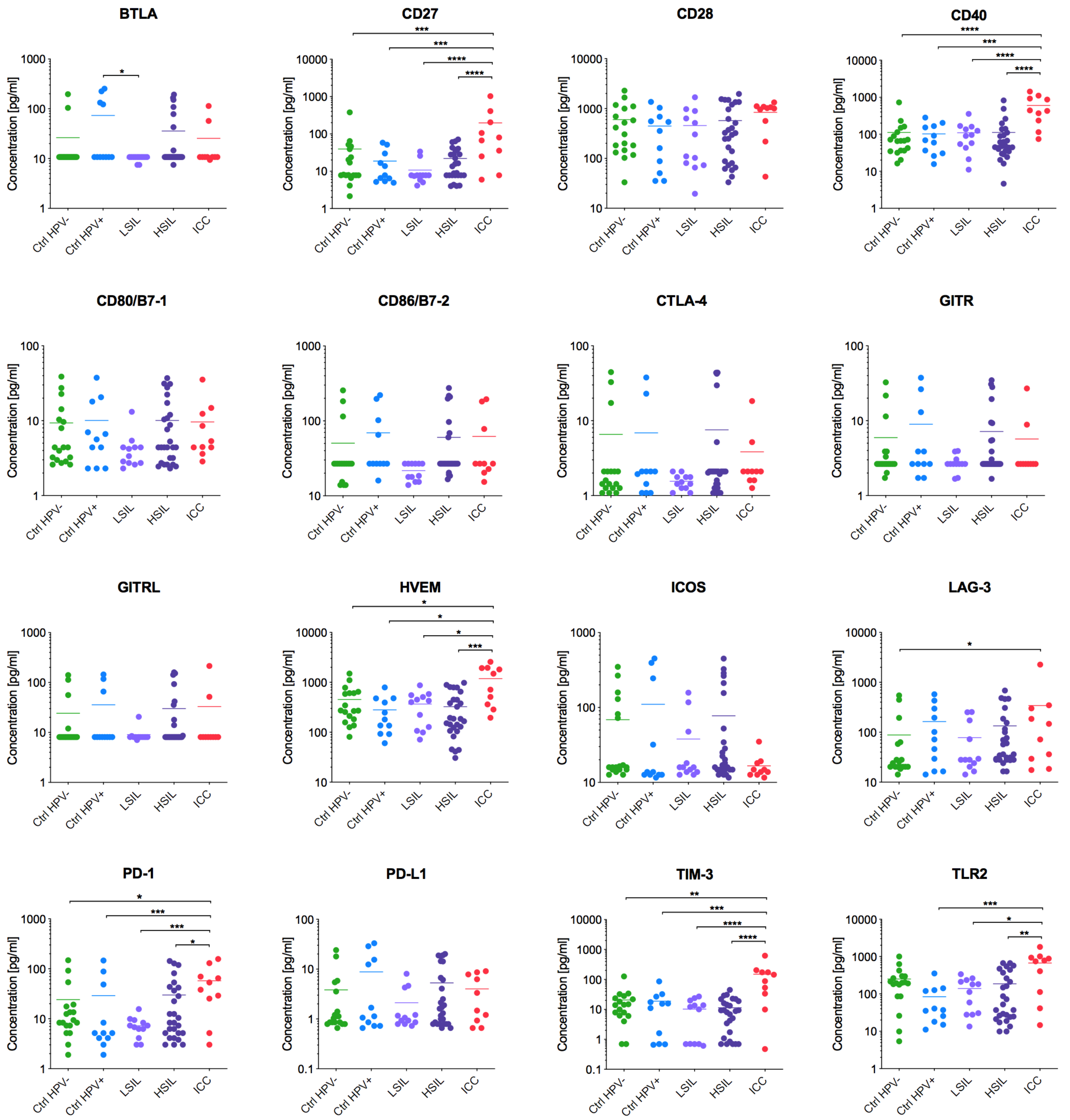

Supplementary Figure S2. Level of immune checkpoint proteins in cervicovaginal lavages in Ctrl HPV–, Ctrl HPV+, LSIL, HSIL and ICC groups. Scatter plots show distribution of protein levels across the groups: healthy HPV-negative controls (Ctrl HPV–), HPV-positive controls (Ctrl HPV+), low-grade squamous intraepithelial lesion (LSIL), high-grade squamous intraepithelial lesion (HSIL) and invasive cervical carcinoma (ICC). Dots indicate individual values for each sample and horizontal solid and dashed lines indicate median and first and third quartiles, respectively. *P* values were calculated using linear mixed effects models where group was the fixed effect and replicate was the random effect with Tukey adjustment. *P* values are indicated with asterisks (**** *P*<0.0001, *** *P*<0.001, ** *P*<0.01, * *P*<0.05).

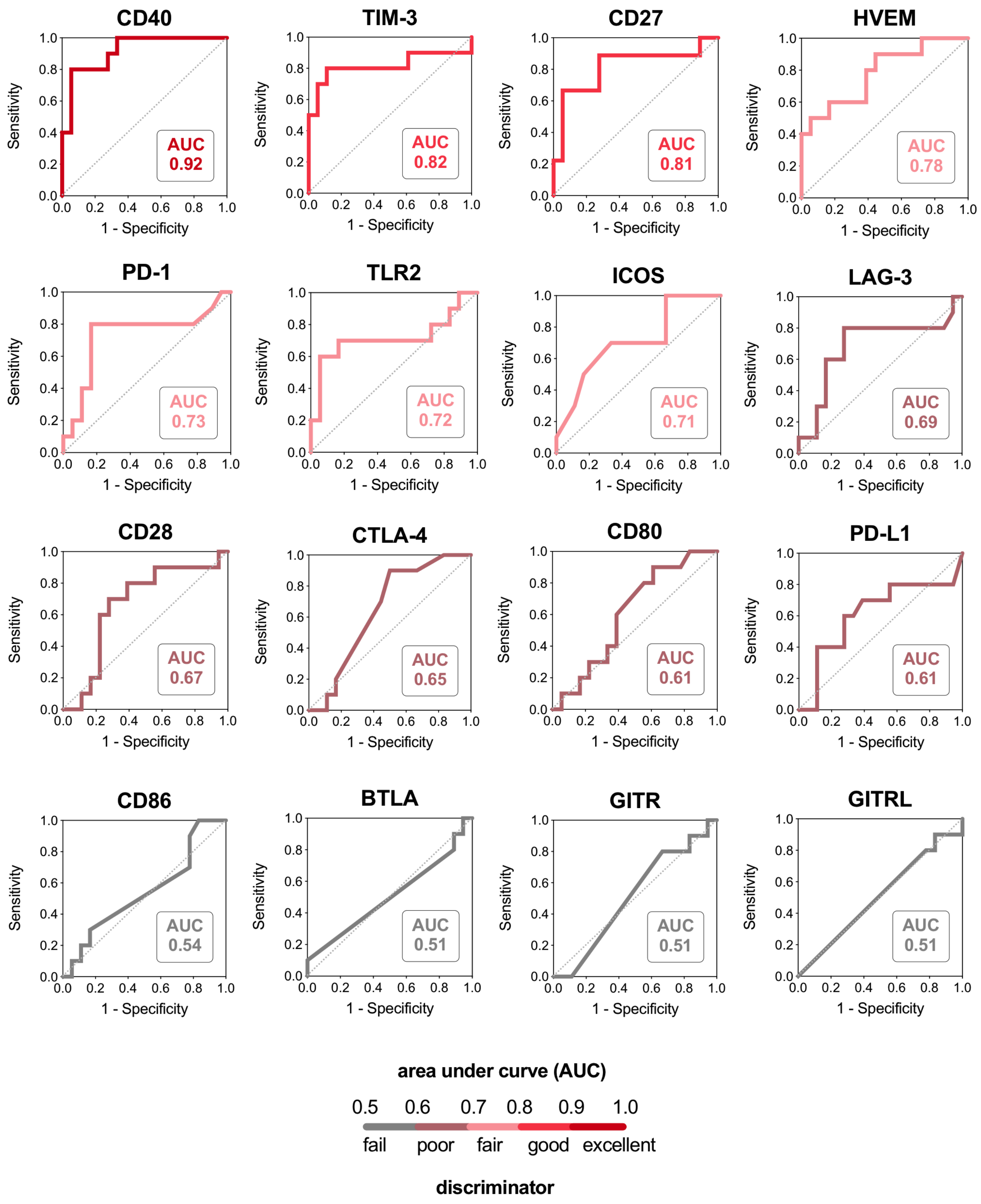

Supplementary Figure S3. Discrimination capacities of immune checkpoint proteins in cervicovaginal lavages for cervical cancer. The receiver operating characteristics (ROC) analysis comparing invasive cervical carcinoma (ICC) to healthy HPV-negative controls (Ctrl HPV–). ROC curves indicate specificity (*x* axis) and 1 – sensitivity (*y* axis). Immune checkpoint proteins with the area under curve (AUC) greater than 0.6, 0.7, 0.8 or 0.9 serve as poor, fair, good or excellent discriminators, respectively. ROC plots are arranged in a decreasing order of AUC values.

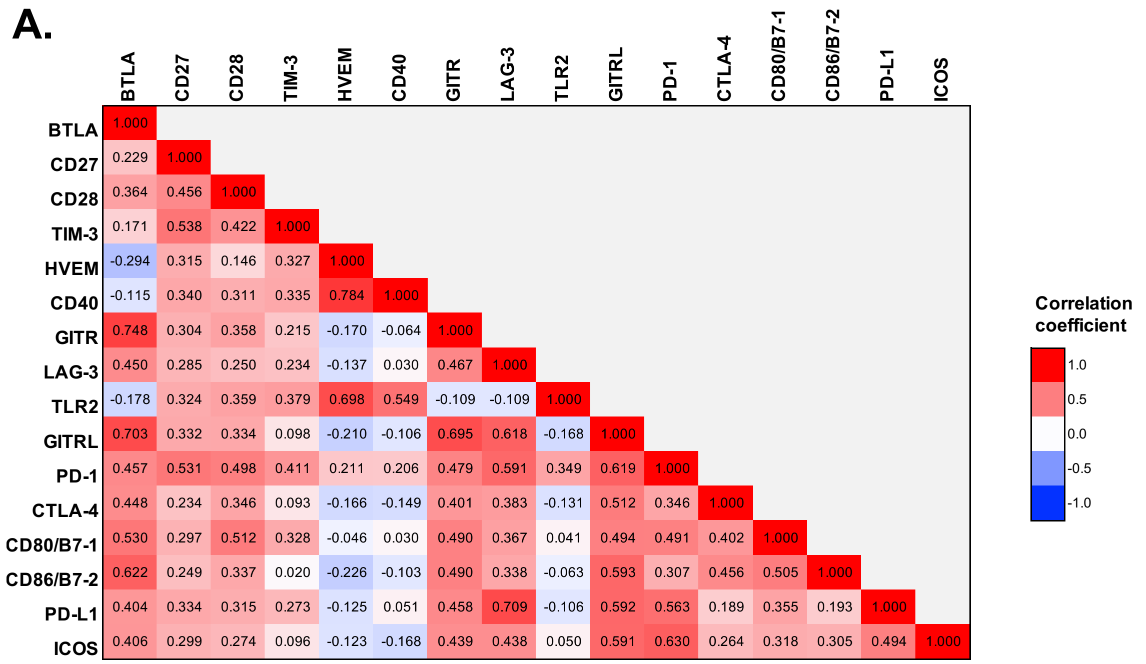

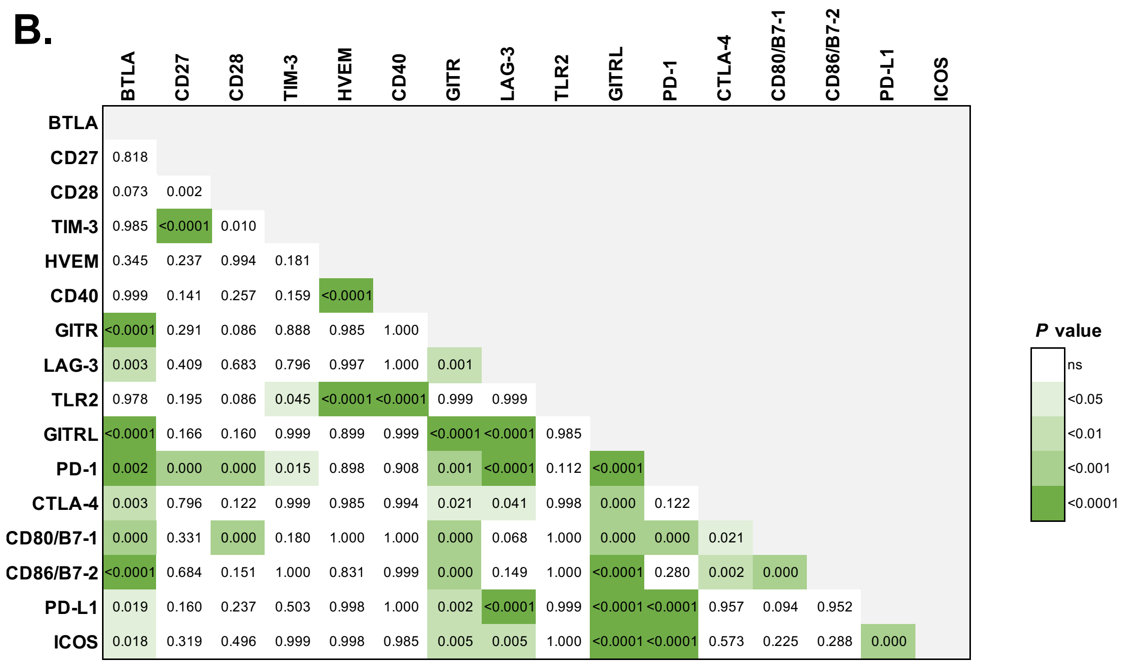

Supplementary Figure S4. Correlation of immune checkpoint proteins to other immune checkpoint proteins in cervicovaginal lavages among all the patients. Correlation coefficients (ρ) were calculated using Spearman's rank correlation analysis. Heat maps shows Spearman's rank correlation coefficients (**A**) or P values (**B**). Red and blue squares indicate positive or negative correlations, respectively, whereas green squares depict different ranges of *P* values.

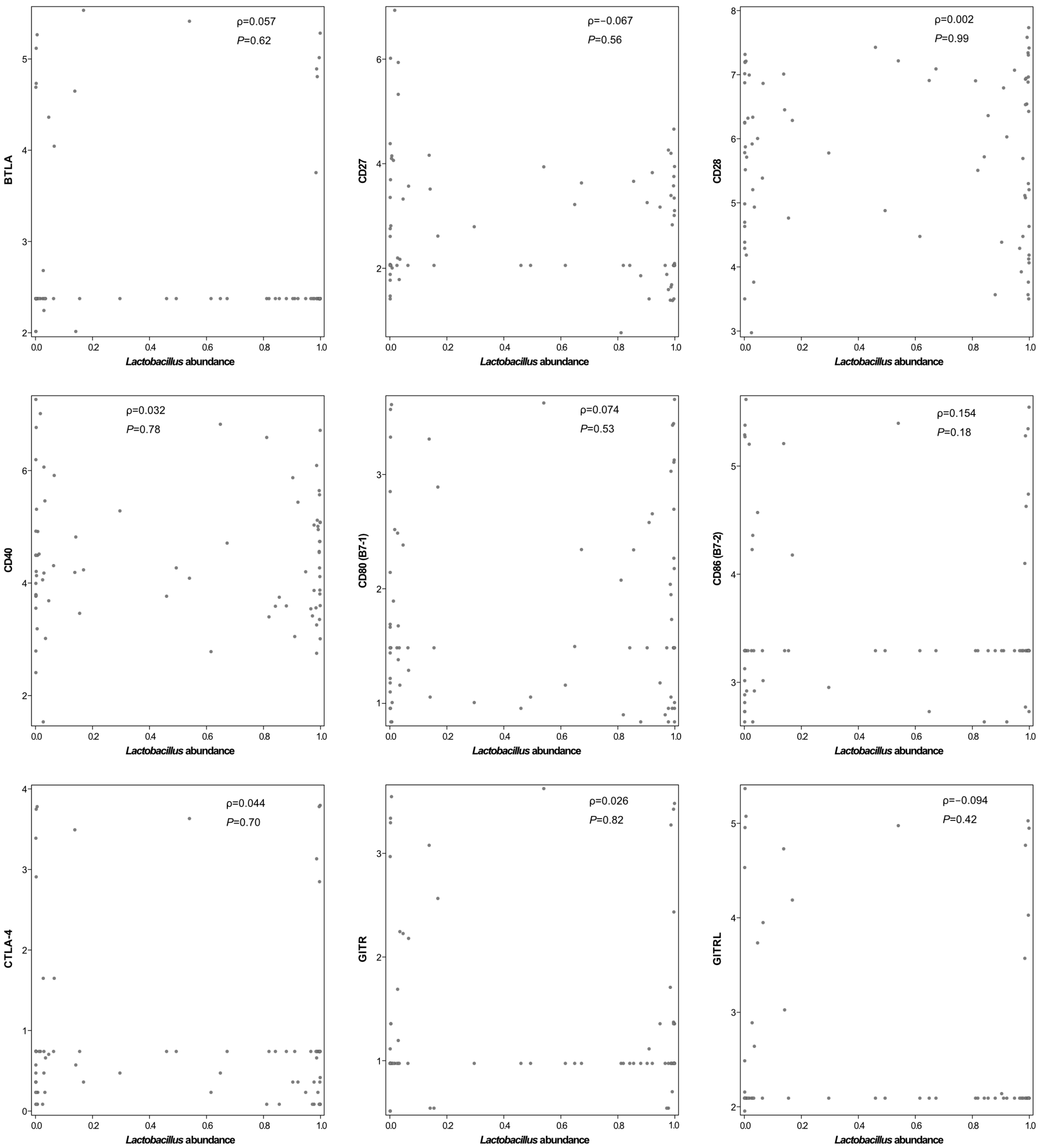
BTLA
ρ=0.057
P=0.62
CD27
ρ=−0.067
P=0.56
CD28
ρ=0.002
P=0.99
CD40
ρ=0.032
P=0.78
CD80 (B7-1)
ρ=0.074
P=0.53
CD86 (B7-2)
ρ=0.154
P=0.18
CTLA-4
ρ=0.044
P=0.70
GITR
ρ=0.026
P=0.82
GITRL
ρ=−0.094
P=0.42
Lactobacillus abundance

cont.

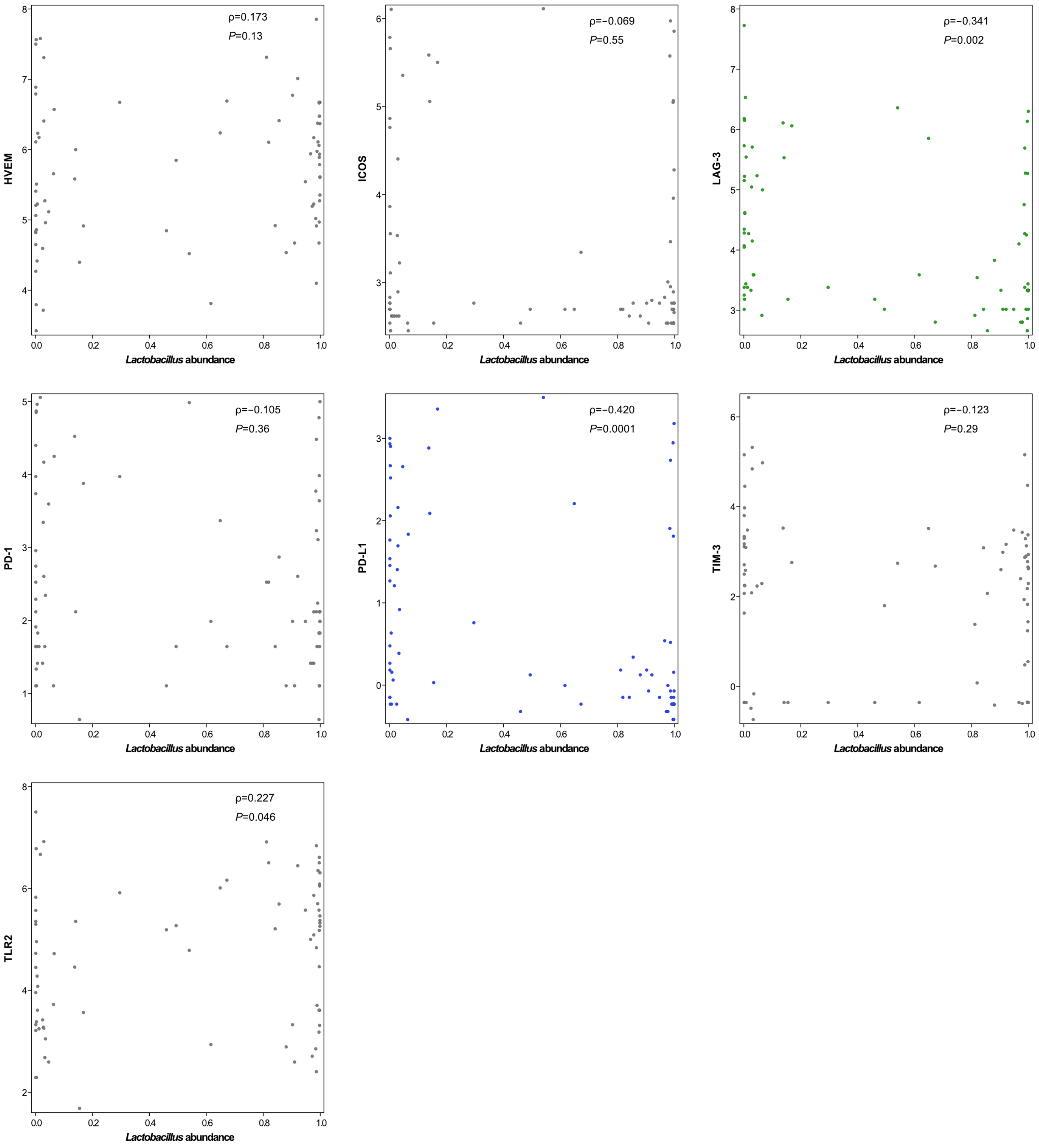

Supplementary Figure S5. Correlations of immune checkpoint proteins with *Lactobacillus* abundance for all samples (n=78). *Lactobacillus* abundance was determined by 16S rRNA gene sequencing. Scatter plots depict correlations between ln-transformed protein levels and *Lactobacillus* abundance for each immune checkpoint protein tested. Correlation coefficients (ρ) were calculated using Spearman's rank correlation analysis. Coefficient and *P* values are shown in the upper right corner of each panel.

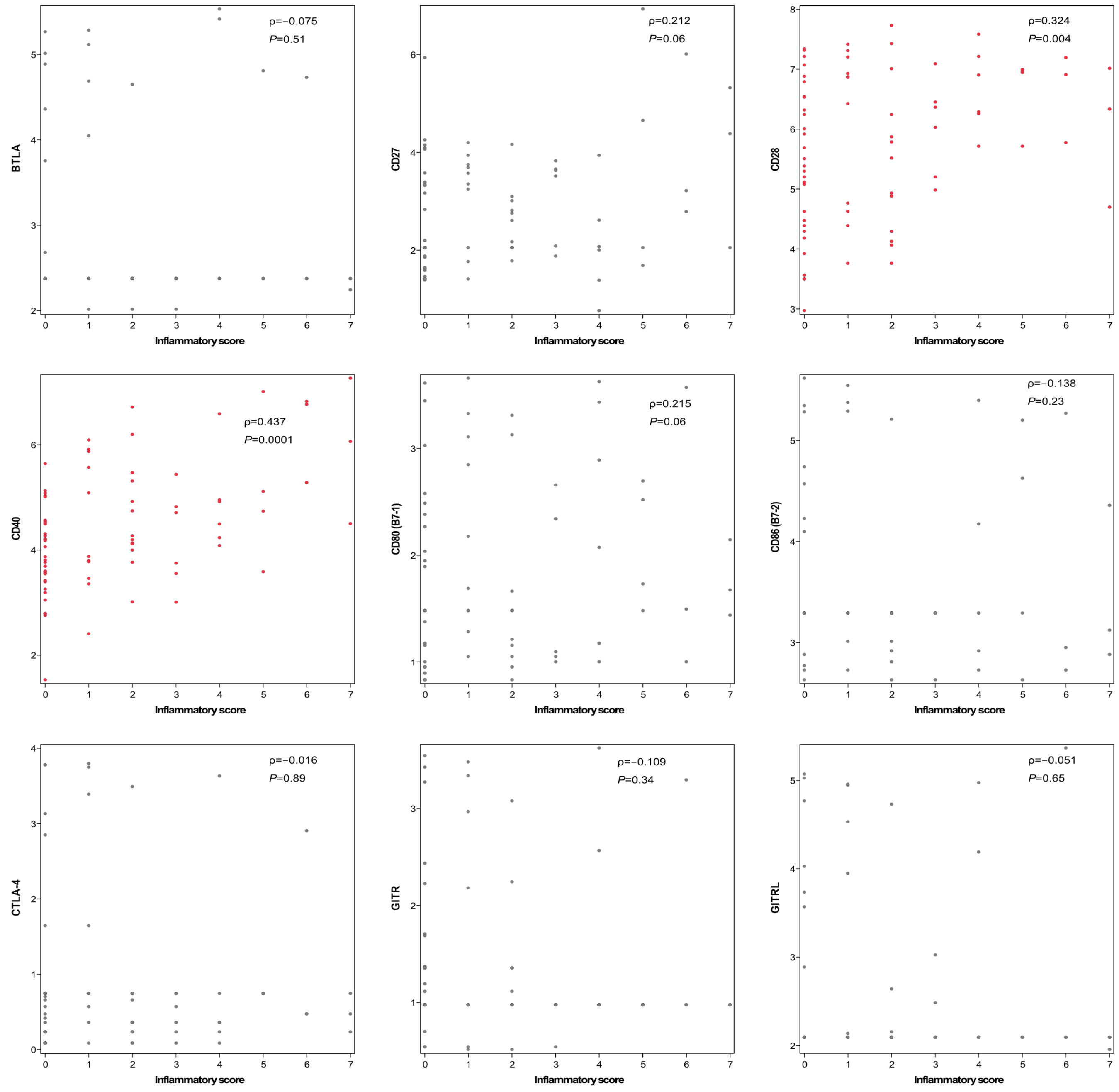

BTLA
ρ=−0.075
P=0.51
Inflammatory score
CD27
ρ=0.212
P=0.06
Inflammatory score
CD28
ρ=0.324
P=0.004
Inflammatory score
CD40
ρ=0.437
P=0.0001
Inflammatory score
CD80 (B7-1)
ρ=0.215
P=0.06
Inflammatory score
CD86 (B7-2)
ρ=−0.138
P=0.23
Inflammatory score
CTLA-4
ρ=−0.016
P=0.89
Inflammatory score
GITR
ρ=−0.109
P=0.34
Inflammatory score
GITRL
ρ=−0.051
P=0.65
Inflammatory score

cont.

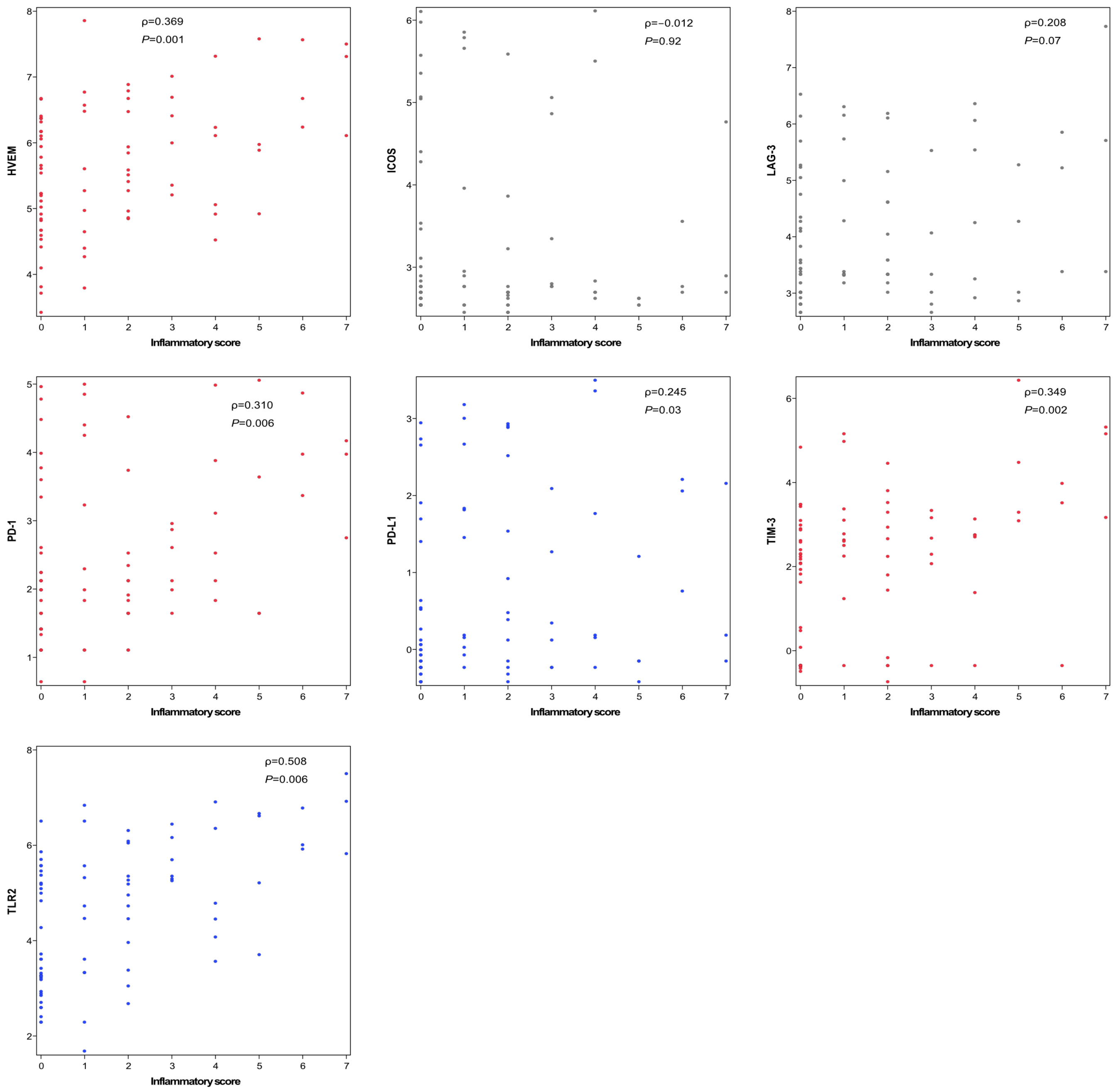

Supplementary Figure S6. Correlations of immune checkpoint proteins with genital inflammatory scores for all samples (n=78). Levels of seven cytokines (IL-1α, IL-1β, IL-8, MIP-1β, MIP-3α, RANTES, TNFα) were evaluated in CVLs and the patients were assigned a genital inflammatory score (0-7) based on whether the level of each cytokine was in the upper quartile. Scatter plots depict correlations between ln-transformed protein levels and genital inflammatory scores for each immune checkpoint protein tested. Correlation coefficients (ρ) were calculated using Spearman’s rank correlation analysis. Coefficient and *P* values are shown in the upper right corner of each panel.

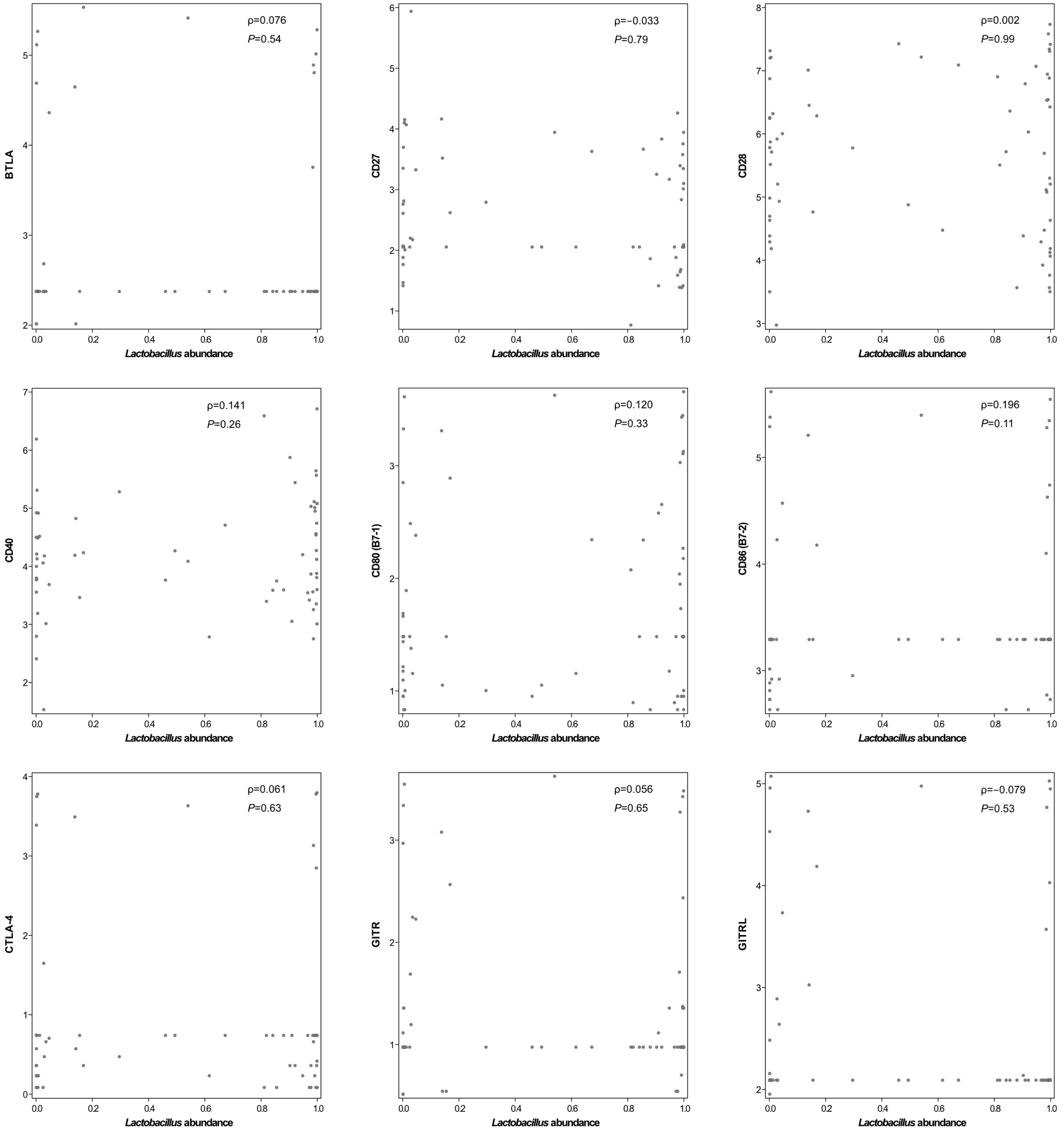
BTLA
ρ=0.076
P=0.54
CD27
ρ=−0.033
P=0.79
CD28
ρ=0.002
P=0.99
CD40
ρ=0.141
P=0.26
CD80 (B7-1)
ρ=0.120
P=0.33
CD86 (B7-2)
ρ=0.196
P=0.11
CTLA-4
ρ=0.061
P=0.63
GITR
ρ=0.056
P=0.65
GITRL
ρ=−0.079
P=0.53
Lactobacillus abundance

cont.

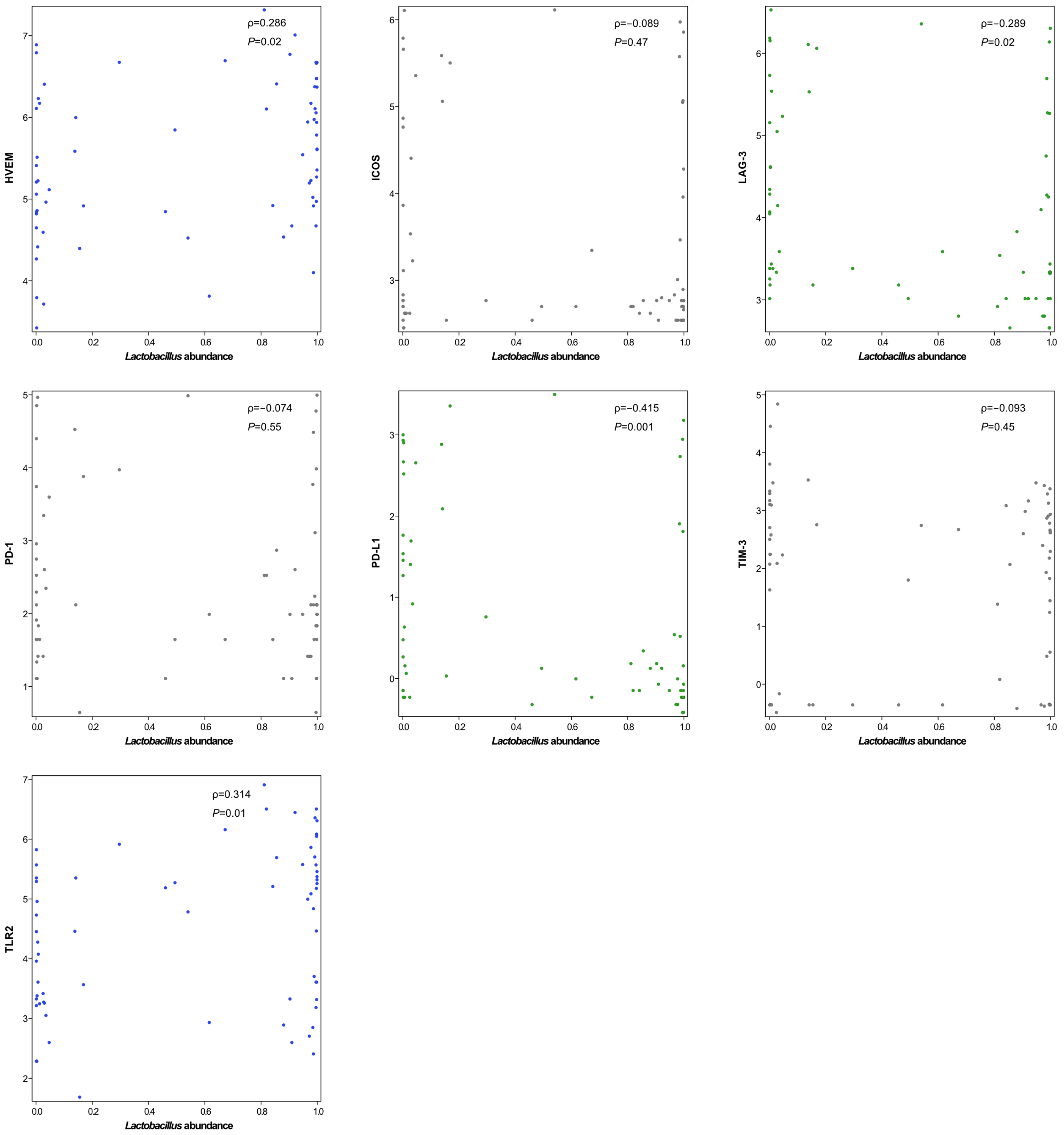

Supplementary Figure S7. Correlations of immune checkpoint proteins with *Lactobacillus* abundance for samples except cancer (n=68). *Lactobacillus* abundance was determined by 16S rRNA gene sequencing. Scatter plots depict correlations between ln-transformed protein levels and *Lactobacillus* abundance for each immune checkpoint protein tested. Correlation coefficients (ρ) were calculated using Spearman's rank correlation analysis. Coefficient and *P* values are shown in the upper right corner of each panel.

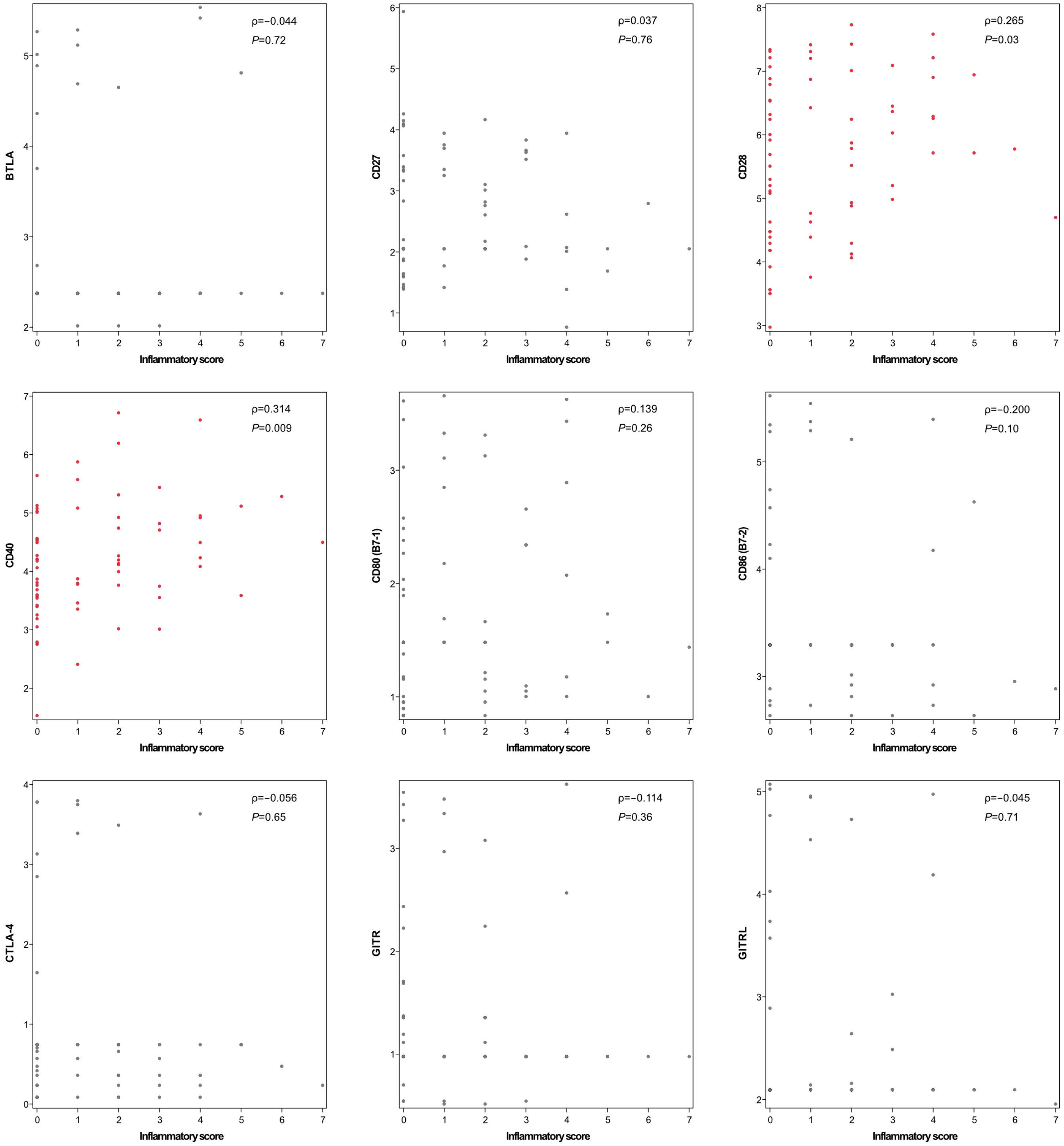
BTLA
ρ=−0.044
P=0.72
CD27
ρ=0.037
P=0.76
CD28
ρ=0.265
P=0.03
CD40
ρ=0.314
P=0.009
CD80 (B7-1)
ρ=0.139
P=0.26
CD86 (B7-2)
ρ=−0.200
P=0.10
CTLA-4
ρ=−0.056
P=0.65
GITR
ρ=−0.114
P=0.36
GITRL
ρ=−0.045
P=0.71
Inflammatory score

cont.

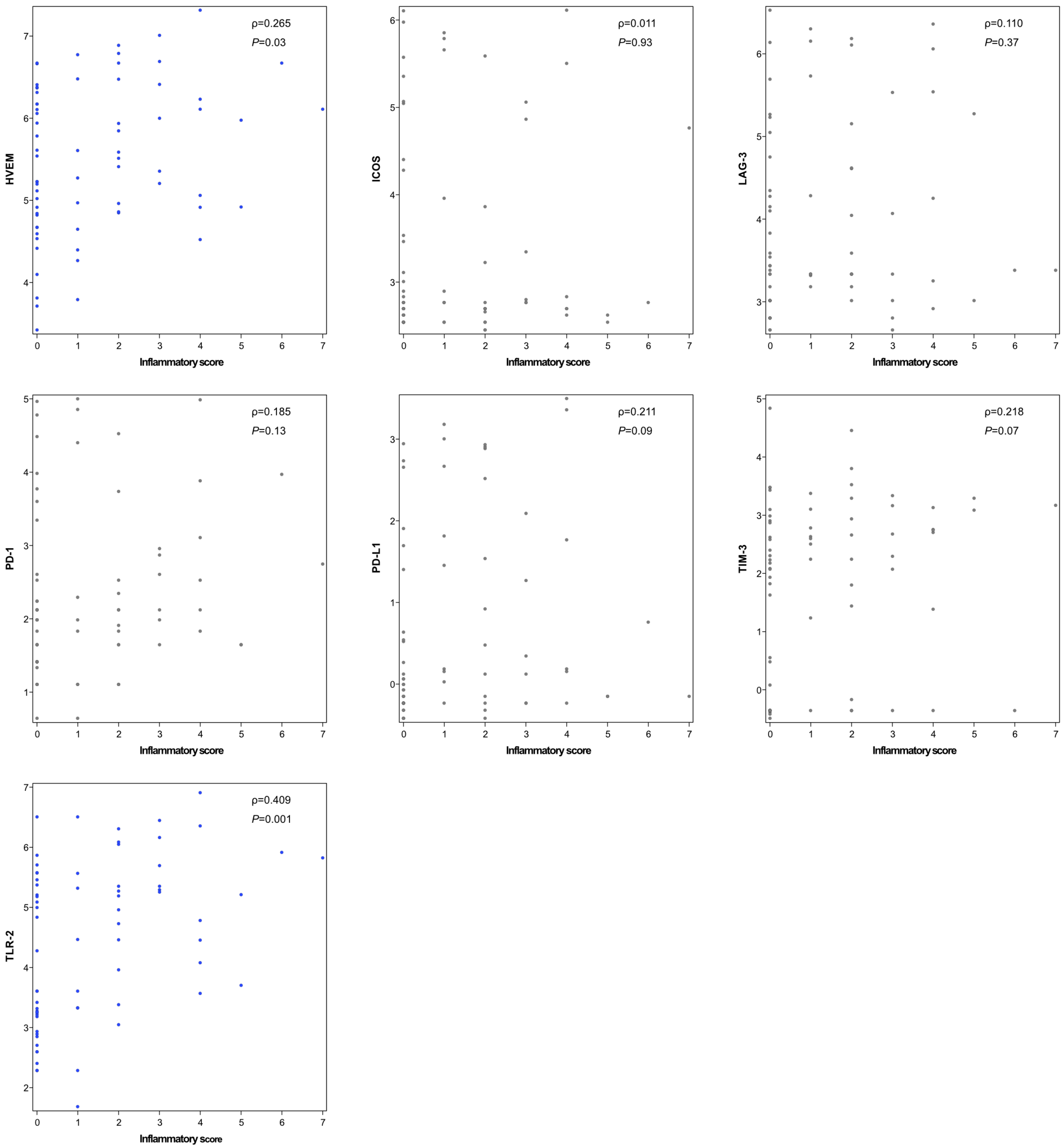

Supplementary Figure S8. Correlations of immune checkpoint proteins with genital inflammatory scores for samples except cancer (n=68). Levels of seven cytokines (IL-1α, IL-1β, IL-8, MIP-1β, MIP-3α, RANTES, TNFα) were evaluated in CVLs and the patients were assigned a genital inflammatory score (0-7) based on whether the level of each cytokine was in the upper quartile. Scatter plots depict correlations between ln-transformed protein levels and genital inflammatory scores for each immune checkpoint protein tested. Correlation coefficients (ρ) were calculated using Spearman's rank correlation analysis. Coefficient and *P* values are shown in the upper right corner of each panel.
